# Biobanking and consenting to research: a qualitative thematic analysis of young people’s perspectives in the North East of England

**DOI:** 10.1186/s12910-023-00925-w

**Published:** 2023-07-05

**Authors:** Fabian J. S. van der Velden, Emma Lim, Lily Gills, Jasmin Broadey, Louise Hayes, Eve Roberts, Jack Courtney, Joanne Ball, Jethro Herberg, Rachel Galassini, Marieke Emonts, DIAMONDS consortium, Michael Levin, Aubrey Cunnington, Myrsini Kaforou, Victoria Wright, Evangelos Bellos, Claire Broderick, Samuel Channon-Wells, Samantha Cooray, Tisham De, Giselle D’Souza, Leire Estramiana Elorrieta, Diego Estrada-Rivadeneyra, Dominic Habgood-Coote, Shea Hamilton, Heather Jackson, James Kavanagh, Mahdi Moradi Marjaneh, Samuel Nichols, Ruud Nijman, Harsita Patel, Ivana Pennisi, Oliver Powell, Ruth Reid, Priyen Shah, Ortensia Vito, Elizabeth Whittaker, Clare Wilson, Rebecca Womersley, Amina Abdulla, Sarah Darnell, Sobia Mustafa, Pantelis Georgiou, Jesus-Rodriguez Manzano, Nicolas Moser, Michael Carter, Shane Tibby, Jonathan Cohen, Francesca Davis, Julia Kenny, Paul Wellman, Marie White, Matthew Fish, Aislinn Jennings, Manu Shankar-Hari, Katy Fidler, Dan Agranoff, Julia Dudley, Vivien Richmond, Matthew Seal, Saul Faust, Dan Owen, Ruth Ensom, Sarah McKay, Diana Mondo, Mariya Shaji, Rachel Schranz, Prita Rughnani, Amutha Anpananthar, Susan Liebeschuetz, Anna Riddell, Divya Divakaran, Louise Han, Nosheen Khalid, Ivone Lancoma Malcolm, Jessica Schofield, Teresa Simagan, Mark Peters, Alasdair Bamford, Lauran O’Neill, Nazima Pathan, Esther Daubney, Debora White, Melissa Heightman, Sarah Eisen, Terry Segal, Lucy Wellings, Simon B. Drysdale, Nicole Branch, Lisa Hamzah, Heather Jarman, Maggie Nyirenda, Lisa Capozzi, Emma Gardiner, Robert Moots, Magda Nasher, Anita Hanson, Michelle Linforth, Sean O’Riordan, Donna Ellis, Akash Deep, Ivan Caro, Fiona Shackley, Arianna Bellini, Stuart Gormley, Samira Neshat, Barnaby J. Scholefield, Ceri Robbins, Helen Winmill, Stéphane C. Paulus, Andrew J. Pollard, Mark Anthony, Sarah Hopton, Danielle Miller, Zoe Oliver, Sally Beer, Bryony Ward, Shrijana Shrestha, Meeru Gurung, Puja Amatya, Bhishma Pokhrel, Sanjeev Man Bijukchhe, Madhav Chandra Gautam, Peter O’Reilly, Sonu Shrestha, Federico Martinón-Torres, Antonio Salas, Fernando Álvez González, Sonia Ares Gómez, Xabier Bello, Mirian Ben García, Fernando Caamaño Viña, Sandra Carnota, María José Curras-Tuala, Ana Dacosta Urbieta, Carlos Durán Suárez, Isabel Ferreiros Vidal, Luisa García Vicente, Alberto Gómez-Carballa, Jose Gómez Rial, Pilar Leboráns Iglesias, Narmeen Mallah, Nazareth Martinón-Torres, José María Martinón Sánchez, Belén Mosquera Perez, Jacobo Pardo-Seco, Sara Pischedda, Sara Rey Vázquez, Irene Rivero Calle, Carmen Rodríguez-Tenreiro, Lorenzo Redondo-Collazo, Sonia Serén Fernández, Marisol Vilas Iglesias, Enital D. Carrol, Elizabeth Cocklin, Abbey Bracken, Ceri Evans, Aakash Khanijau, Rebecca Lenihan, Nadia Lewis-Burke, Karen Newall, Sam Romaine, Jennifer Whitbread, Maria Tsolia, Irini Eleftheriou, Nikos Spyridis, Maria Tambouratzi, Despoina Maritsi, Antonios Marmarinos, Marietta Xagorari, Lourida Panagiota, Pefanis Aggelos, Akinosoglou Karolina, Gogos Charalambos, Maragos Markos, Voulgarelis Michalis, Stergiou Ioanna, John Isaacs, Kathryn Bell, Stephen Crulley, Daniel Fabian, Evelyn Thomson, Diane Walia, Caroline Miller, Ashley Bell, Geoff Shenton, Ashley Price, Owen Treloar, Daisy Thomas, Pablo Rojo, Cristina Epalza, Serena Villaverde, Sonia Márquez, Manuel Gijón, Fátima Marchín, Laura Cabello, Irene Hernández, Lourdes Gutiérrez, Ángela Manzanares, Taco W. Kuijpers, Martijn van de Kuip, Marceline van Furth, Merlijn van den Berg, Giske Biesbroek, Floris Verkuil, Carlijn W. van der Zee, Dasja Pajkrt, Michael Boele van Hensbroek, Dieneke Schonenberg, Mariken Gruppen, Sietse Nagelkerke, Machiel H. Jansen, Ines Goedschalckx, Lorenza Romani, Maia De Luca, Sara Chiurchiù, Constanza Tripiciano, Stefania Mercadante, Clementien L. Vermont, Henriëtte A. Moll, Dorine M. Borensztajn, Nienke N. Hagedoorn, Chantal Tan, Joany Zachariasse, Willem A. Dik, Shen Ching-Fen, Dace Zavadska, Sniedze Laivacuma, Aleksandra Rudzate, Diana Stoldere, Arta Barzdina, Elza Barzdina, Monta Madelane, Dagne Gravele, Dace Svile, Romain Basmaci, Noémie Lachaume, Pauline Bories, Raja Ben Tkhayat, Laura Chériaux, Juraté Davoust, Kim-Thanh Ong, Marie Cotillon, Thibault de Groc, Sébastien Le, Nathalie Vergnault, Hélène Sée, Laure Cohen, Alice de Tugny, Nevena Danekova, Marine Mommert-Tripon, Marko Pokorn, Mojca Kolnik, Tadej Avčin, Tanja Avramoska, Natalija Bahovec, Petra Bogovič, Lidija Kitanovski, Mirijam Nahtigal, Lea Papst, Tina Plankar Srovin, Franc Strle, Katarina Vincek, Michiel van der Flier, Wim J. E. Tissing, Roelie MWösten-van Asperen, Sebastiaan J. Vastert, Daniel C. Vijlbrief, Louis J. Bont, Tom F. W. Wolfs, Coco R. Beudeker, Sanne C. Hulsmann, Philipp K. A. Agyeman, Luregn Schlapbach, Christoph Aebi, Mariama Usman, Stefanie Schlüchter, Verena Wyss, Nina Schöbi, Elisa Zimmermann, Marion Meier, Kathrin Weber, Eric Giannoni, Martin Stocker, Klara M. Posfay-Barbe, Ulrich Heininger, Sara Bernhard-Stirnemann, Anita Niederer-Loher, Christian Kahlert, Giancarlo Natalucci, Christa Relly, Thomas Riedel, Christoph Berger, Colin Fink, Marie Voice, Leo Calvo-Bado, Michael Steele, Jennifer Holden, Andrew Taylor, Ronan Calvez, Catherine Davies, Benjamin Evans, Jake Stevens, Peter Matthews, Kyle Billing, Werner Zenz, Alexander Binder, Benno Kohlmaier, Daniel S. Kohlfürst, Nina A. Schweintzger, Christoph Zurl, Susanne Hösele, Manuel Leitner, Lena Pölz, Alexandra Rusu, Glorija Rajic, Bianca Stoiser, Martina Strempfl, Manfred G. Sagmeister, Sebastian Bauchinger, Martin Benesch, Astrid Ceolotto, Ernst Eber, Siegfried Gallistl, Harald Haidl, Almuthe Hauer, Christa Hude, Andreas Kapper, Markus Keldorfer, Sabine Löffler, Tobias Niedrist, Heidemarie Pilch, Andreas Pfleger, Klaus Pfurtscheller, Siegfried Rödl, Andrea Skrabl-Baumgartner, Volker Strenger, Elmar Wallner, Maike K. Tauchert, Ulrich von Both, Laura Kolberg, Patricia Schmied, Ioanna Mavridi, Irene Alba-Alejandre, Katharina Danhauser, Niklaus Haas, Florian Hoffmann, Matthias Griese, Tobias Feuchtinger, Sabrina Juranek, Matthias Kappler, Eberhard Lurz, Esther Maier, Karl Reiter, Carola Schoen, Sebastian Schroepf, Shunmay Yeung, Manuel Dewez, David Bath, Elizabeth Fitchett, Fiona Cresswell, Effua Usuf, Kalifa Bojang, Anna Roca, Isatou Sarr, Momodou Ndure

**Affiliations:** 1 Paediatric Immunology, Infectious Diseases & Allergy, Great North Children’s Hospital, The Newcastle Upon Tyne Hospitals NHS Foundation Trust, Newcastle Upon Tyne, UK; 2 Translational and Clinical Research Institute, Newcastle University, Newcastle Upon Tyne, UK; 3 Population Health Sciences Institute, Newcastle University, Newcastle Upon Tyne, UK; 4 General Paediatrics, Great North Children’s Hospital, The Newcastle Upon Tyne Hospitals NHS Foundation Trust, Newcastle Upon Tyne, UK; 5 Young Person’s Advisory Group North England, Great North Children’s Hospital, The Newcastle Upon Tyne Hospitals NHS Foundation Trust, Newcastle Upon Tyne, UK; 6 The Great North Youth Forum, Great North Children’s Hospital, The Newcastle Upon Tyne Hospitals NHS Foundation Trust, Newcastle Upon Tyne, UK; 7 Section of Paediatric Infectious Disease, Wright-Fleming Institute, Imperial College London, London, UK

**Keywords:** Paediatrics, Biobank, Consent, Assent, Children and young people

## Abstract

**Background:**

Biobanking biospecimens and consent are common practice in paediatric research. We need to explore children and young people’s (CYP) knowledge and perspectives around the use of and consent to biobanking. This will ensure meaningful informed consent can be obtained and improve current consent procedures.

**Methods:**

We designed a survey, in co-production with CYP, collecting demographic data, views on biobanking, and consent using three scenarios: 1) prospective consent, 2) deferred consent, and 3) reconsent and assent at age of capacity. The survey was disseminated via the Young Person’s Advisory Group North England (YPAGne) and participating CYP’s secondary schools. Data were analysed using a qualitative thematic approach by three independent reviewers (including CYP) to identify common themes. Data triangulation occurred independently by a fourth reviewer.

**Results:**

One hundred two CYP completed the survey. Most were between 16–18 years (63.7%, *N* = 65) and female (66.7%, *N* = 68). 72.3% had no prior knowledge of biobanking (*N* = 73).

Acceptability of prospective consent for biobanking was high (91.2%, *N* = 93) with common themes: ‘altruism’, ‘potential benefits outweigh individual risk’, 'frugality', and ‘(in)convenience’.

Deferred consent was also deemed acceptable in the large majority (84.3%, *N* = 86), with common themes: ‘altruism’, ‘body integrity’ and ‘sample frugality’. 76.5% preferred to reconsent when cognitively mature enough to give assent (*N* = 78), even if parental consent was previously in place. 79.2% wanted to be informed if their biobanked biospecimen is reused (*N* = 80).

**Conclusion:**

Prospective and deferred consent acceptability for biobanking is high among CYP in the UK. Altruism, frugality, body integrity, and privacy are the most important themes. Clear communication and justification are paramount to obtain consent. Any CYP with capacity should be part of the consenting procedure, if possible.

**Supplementary Information:**

The online version contains supplementary material available at 10.1186/s12910-023-00925-w.

## Introduction

Biobanking has become increasingly common, in biomedical research, and within in paediatrics [1, 2]. Biobanks are valuable repositories of human biospecimens, associated data and technological infrastructure [3]. Biobanks provide an important resource for biomedical research in the development of new diagnostic methods, treatments, determinants of disease in various contexts.

The success of a biobank relies on the willingness of people to donate samples, and incurs many ethical, social and legal challenges. Biobanking raises unique issues with regards to consenting procedures, sample donation, data confidentiality and privacy [4–6]. There is little guidance around the ethics of biobanking, however the Council for International Organizations of Medical Sciences (CIOMS) has published guidelines [7], addressing issues around collection, storage, and use of biological materials and related data in health-related research. They provide general and specific considerations for biobanks, including governance for keeping participants informed of research outcomes, consent, withdrawal of consent and opt out procedures, and unexpected or unsolicited findings alongside the storage and use of genetic material. Ashcroft and Macpherson [6] identified further ethical concerns including *“misconceptions about biobanking and distinctions between research, diagnostics, and treatment; unknown consequences of, and harms to, individual and collective donors of materials or information and socioeconomic inequities that impinge on donor understanding and voluntariness and increase their vulnerabilities to harms and wrongs”.* The complex nature of obtaining informed consent in diverse cultural and socioeconomic context, especially during public health emergencies is particularly difficult. Whilst these issues are under ethical debate, there is currently no definite consensus opinion, especially for children and young persons [8]. Children and, to a lesser extent, young persons lack the capacity to consent, therefore, the ethical consensus in adult research cannot be extrapolated to paediatrics. The Mental Capacity Act [9] applies to children who are 16 years and over. *“Mental capacity is present if a person can understand information given to them, retain the information given to them long enough to make a decision, can weigh up the advantages and disadvantages of the proposed course of treatment in order to make a decision, and can communicate their decision.”* [9, 10]. Once a young person is sixteen years old, they are deemed competent to consent or refuse treatment and their parents cannot override them. For children under sixteen years of age the Act does not apply and they need to be assessed for ‘Gillick competence’. This a term used in law to decide if a child is competent to consent for their own medical treatment, without parental permission or knowledge Parents cannot override a competent child’s refusal to accept treatment. Gillick competency is a professional assessment and there is no set of defined questions [11]. It is often used in a wider context to help assess whether a child has the maturity to make their own decisions and to understand the implications of those decisions.

Prospective consent is the golden standard of consent. As children cannot legally consent, this is sought from parents or their legal guardian [8]. Obtaining assent from the child or young person (CYP) is deemed best clinical practice.

In 2008 the United Kingdom (UK) amended legislation allowing deferred consent for research in an emergency setting [12]. Collecting samples in an emergency allows increased access to quality samples (for example before treatments are given) but also allows samples to be taken at the same time as clinically necessary tests which reduces the number of potentially invasive or painful tests (blood sampling) and the distress that can cause for CYP.

Deferred consent addresses difficulties encountered whilst conducting research in emergency settings [13], mainly those related to time constraints for sample collection and parental capacity in a stressful setting [14, 15]. Others argue deferred consent interferes with core values of informed consent, represents a dishonest attempt to justify recruitment without consent, and compromises autonomy [14].

Further ethical issues involve reconsent. As children grow up, most children developmentally move from minimal to robust autonomy in adolescence, and develop the capacity to consent [16]. Biobanked samples might be stored and used for decades, by which time the CYP will have developed capacity, and may have new insights regarding use of their donated samples and associated personal data [4]. This suggests that reconsent may be necessary to justify use of the samples beyond childhood.

Although aforementioned ethical issues are under debate, the literature mainly focuses on the theoretical debate [4, 17, 18] or involves perspectives of parents or legal guardians [5, 19, 20], practitioners [15], the critically ill [14, 21] or adolescents already participating in research involving biobanks [22, 23]. Perspectives of the general population mainly involves adults, with university students being closest to the paediatric population [24, 25]. Literature on the CYP’s perspective is surprisingly sparse, despite being fundamental to the ongoing ethical debate.

This study aimed to assess CYP perspectives in the community towards prospective consent, deferred consent, reconsent and assent for biobanked samples. Secondary, we investigated views on sample donation, donation hesitation and data handling.

## Methods

### Study design and ethics

This was a cross-sectional, qualitative survey-based study. Anonymity of participants was maintained throughout the entire study. As part of the Diagnosis and Management of Febrile Illness using RNA Personalised Molecular Signature Diagnosis (DIAMONDS) study, ethics approval was obtained for the UK under: IRAS 209035, REC 16/LO/1684. DIAMONDS aims to establish a biobank of host gene signatures of common inflammatory and infectious causes of febrile disease in children. The survey was voluntary and anonymous. Informed consent was obtained from all participants. Written informed consent for the survey from parents or legal guardians was not required or obtained for patients under 16 years of age, with approval from the Newcastle and North Tyneside Research Ethics Committee. The survey was registered with our local audit registry, under audit number 13906.

Parental input was seen as a barrier to CYP participation and would potentially influence the responses. The co-produced element of the project ensured the subject and information created was CYP friendly, which significantly impacts their capacity to understand the circumstances and details of the research being proposed. The information was written and presented in a CYP friendly manner, to ensure CYP had all the information required to consent. Gillick competence was applied: reading the introductory information paragraph preceding the survey and the ability to complete the survey constituted consent for participants under the age of 16 years.

### Participants

The target population were CYP attending secondary schools in the North East of England. Any CYP aged 11–21 years inclusive involved with the Young Person’s Advisory Group North England (YPAGne) was invited to complete the survey, and they recruited further CYP who were not YPAGne members from their respective secondary schools. The participants did not donate samples to the DIAMONDS study and they were specifically asked to look independently at the ethical implications of biobanking biospecimens.

### Survey development

The tailored survey was designed by volunteer CYP co-opted from YPAGne together with experienced researchers. YPAGne (https://sites.google.com/nihr.ac.uk/ypagne) is an independent organisation within our hospital that exists to promote youth voice in health research and involve them in a variety of research projects from conception until the end. The co-opted CYP who designed the survey voluntarily chose to work on the biobanking project out of their own interest. The Youth Collective Engagement Coordinator (JBa), who is not medically trained, facilitates the projects, ensures impartiality and aims to maximise the CYP’s skill development through research projects. The DIAMONDS team approached YPAGne to conduct this survey project on CYP perspectives regarding biobanking.

The CYP undertook a literature review and met with independent researchers with extensive biobanking knowledge to gain more knowledge on the topic of biobanking.

Subsequently the co-opted CYP developed the hypothetical case scenarios and the survey questions, in multiple sessions. At this stage researchers in the DIAMONDS consortium were not yet involved, to ensure survey development was neutral and not affected by any potential bias induced by the research team.

A pilot was conducted, within the wider YPAGne forum, to provide feedback on the suitability and clarity of the survey. In a facilitated group session, minor semantic modifications were made in consultation with the DIAMONDS research team, and the final version of the survey approved. There were no major modifications on the content of the survey, as the research team was in agreement with the proposed survey. After some debate, a paragraph explaining what the purpose of biobank is, was added to the survey. This was felt necessary to ensure CYP in the community with no prior knowledge of biobanking could meaningfully complete the survey.

The final survey consisted of three sections, preceded by an introductory page explaining the purpose of the study, biobanking, anonymous and voluntary participation. Survey completion was regarded as agreement to participation and consent.

The first section covered demographic data. The second section included case-based questions regarding prospective consent, deferred consent, and reconsent and assent. The final section assessed attitudes towards sample donation and data confidentiality.

### Data collection

The final survey (Additional File 1) was created using Google Forms (http://docs.google.com/forms/). The weblinks for the survey were distributed by CYP via their schools’ email service, YPAGne’s Facebook page, and other social media platforms such as WhatsApp, Instagram, Twitter and Snapchat. Data was collected from February to April 2021, with a second survey distribution in December 2021 to achieve data saturation.

### Measures

#### Demographics

Participants were asked their age (in groups), self-identified gender (male, female, prefer not to say, other), partial postcodes, previous knowledge of biobanking (yes/no), and any previous hospital admission (yes/no).

#### Case-based consent procedures for biobanking

Case 1 revolved around prospective informed consent. We presented a case of a CYP attending for routine medical procedures and being asked to donate an extra sample for a biobank. Case 2 involved deferred consent. We described a case in which CYP had blood taken for a diagnostic test in an emergency and an extra blood sample preserved for biobanking. Consent was asked retrospectively with an option for the sample to be destroyed if consent was not given. Attitudes regarding prospective and deferred consent were measured on a 4-point scale ranging from ‘strongly disagree’ to ‘strongly agree’. Subsequently, participants were asked to explain their answer in an open-ended question.

In case 3, we explored when a parent or legal guardian consented to a CYP having a blood sample stored in a biobank for research, if the participant would want to be asked at a later age whether they would give permission for their sample to be biobanked. If they wanted this, we asked at what age, and their opinions on reconsent or assent.

Lastly, we asked if the participant felt that CYP should be involved in the consent procedure whenever possible, and why.

#### Sample donation and data handling﻿

We asked what kind(s) of biospecimens participants would be comfortable with donating to a biobank if they were collected for medical or surgical procedures regardless, followed by a question about why they would not be comfortable donating any samples. We also asked participants what information they would feel comfortable with to be stored alongside their biobanked specimens, and why they might be hesitant to donate samples to a biobank. To conclude the survey, they were asked about preferences regarding being informed when their sample is used, how they want to find out and what they want to know about sample use.

### Data analysis

Quantitative data from closed questions were analysed in SPSS version 27 [Armonk USA 2020].

Qualitative data from open-ended questions were analysed using a qualitative thematic approach. Three reviewers independently analysed the open questions.

The first group of reviewers consisted of two CYP working on our study (LG and JBr) with the assistance of an experienced researcher (LH). In separate meetings they explored the data identified common themes. One meeting each was dedicated to prospective consent, deferred consent, reconsent, or donation hesitation and data confidentiality. The second (FvdV) and third (EL) reviewers identified common themes on these subjects independently. The fourth reviewer (JC) triangulated the analyses from the three reviewers to enable independent reporting and data saturation [26].

## Results

One hundred two CYP completed the survey. The majority of participants were female (66.7%, *N* = 68), from North East England and Cumbria (86.2%, *N* = 88), between 16–18 years old (63.7%, *N* = 65), and had no previous knowledge of biobanking (72.3%, *N* = 74). Table 1 shows the demographic characteristics of the participants, and Table 2 gives an overview of quotes from participants on the different consent approaches.

**Table 1:** Demographic characteristics of CYP in survey (*N* = 102)

Characteristic	*N* = (%)
**Gender (self-identified)**	
Female	68 (66.7%)
Male	26 (25.5%)
Non-binary	1 (1.0%)
Agender	1 (1.0%)
Prefer not to say	6 (5.8%)
**Age**	
11 years	1 (1.0%)
12–15 years	13 (12.8%)
16–18 years	65 (63.7%)
≥ 18 years	23 (22.5%)
**Geographical region**	
North East England and Cumbria	88 (86.2%)
Greater London	11 (10.8%)
South West England	3 (3.0%)
**Previous knowledge of biobanking**	
Yes	28 (27.5%)
No	74 (72.5%)
**Previous admission to hospital (overnight)**	
Yes	41 (40.2%)
No	61 (59.8%)

**Table 2:** Illustrative CYP quotes by consent strategy for biobanking and identified common themes

Consent strategy	Quote
**Prospective consent**	
Altruism	*‘Medical research is very important, and I would not mind helping’* *‘It allows the NHS to help more people who need it more than me’*
Benefits outweigh risks	*‘There is no difference having extra taken, when the good it can do is immense’* *‘I have been asked for consent, and the procedure is happening anyways, so no harm to take it’*
Frugality	*‘It would be a shame for any blood or tissue to go to waste when it could help someone’*
(In)convenience	*‘It depends on what they’re taking and if I feel comfortable with it’* *‘It might depend on whether it would have noticeable impact on me/recovery time’*
**Deferred consent**	
Altruism	*‘Happy [to donate] if there is something left that could be helpful’* *‘It has no effect on me, yet has benefits for other people’*
Body integrity	*‘I’m not entirely sure on this one: as long as consent is asked after it should be okay, but I also don’t like the idea of something being done to my body without consent in advance’* *‘I think deferred consent is wrong and I would definitely feel violated in this case’*
Frugality	*‘I think destroying the sample would be a waste so it should go to a biobank’* *‘If the blood had been taken, it would better be used for something, rather than just being destroyed’*
**Assent and Reconsent**	
Ownership	*‘I think children deserve the right to consent, as long as they understand the choice they are making* *‘A child’s no should be able to override a parents’ yes. But a child’s yes should require a yes from the parents as well’* *‘I think parents should make most of the decisions for young children, but definitely talk to them about it and ask how they feel’*

### Prospective consent

Most participants 91.2% (*N* = 93) had a positive attitude towards prospective consent for biobanking: 46 agreed and 47 strongly agreed. Of the 9 participants with negative attitudes (8.8%), only 1 strongly disagreed with prospective consent to biobanking.

Altruistic reasons for participation were frequently expressed in statements such as: “*I think the research done on it will be good for future patients*” and “*To take [from the health care system] you should be willing to give*”.

Participants indicate potential benefits of specimen donation, outweigh the risk of the additional procedure. “*I would feel happy about giving an extra blood sample. I would agree to a tissue sample if it would not leave side effects or much extra scarring”*.

They did voice important considerations and would not necessarily agree to donating without clear communication. Participants want to know how and what the sample is used for: “*I would be content as long as it goes to a good cause*” or “*I’d be happy with additional samples to be taken as extra to the procedure being done given an informative explanation*”. They also highlighted the importance of being given information in advance, stating “*I would be fine with it, but would require a bit of time beforehand; just so I am fully aware of what I’m doing*”.

The most common reasons for those less positive about biobanking were: needle phobia, perceived additional pain related to the procedure or concerns about how the sample will be used.

### Deferred consent

Deferred consent was highly acceptable among participants (84.3%): 18 agreed, and 68 strongly agreed. Only five strongly disagreed with deferred consent for biobanking (4.9%).

We observed a shift in motivation with less of a focus on altruism, but more towards frugality (Table 1). Participants realise and accept, in emergency situations, samples may be taken prior to consent being obtained. The majority of participants stated opinions such as *“They would not use my sample for anything until I had given consent, so the choice is still in the hands of the patient*” and “*I really don’t mind. If it’s an emergency situation it’s clearly inappropriate to ask at the time, so asking afterwards is fine. Again, as long as it has no effect on my care, take what you need*”. Others feel more uncomfortable but would still agree: “*I would be quite annoyed if they [take a sample for biobanking] without consent, but I’d also be happy for it to be used”.* A few disagreed with the concept and one person stated how this impacted their autonomy and dignity: *“I think deferred consent is wrong and I would definitely feel violated in this case”*.

In cases where consent is refused, participants described the importance of trust and transparency regarding sample handling. “*I think it is important to provide reassurance and information about how the sample will be destroyed to ensure patients who did not give consent do not need to worry about the hospital keeping the sample”*.

### Assent and reconsent

All participants thought CYP should be involved in the consent procedure, “*Children still have rights and I think it’s important that they feel included in decisions about their own body and medical care*”*.* Participants thought CYP should have the ‘power of veto’ over donating samples “*A* child's no *should be able to override a parents* yes*. But a child's* yes *should require* a yes *from the parents as well*”.

Three quarters of participants (76.5%, *N* = 78) wanted to be contacted at an older age to reconsent for sample use, if they had samples taken before the age of capacity but a quarter (24.5%, *N* = 25) did not feel they needed to be contacted again. With regards to age of reconsent, 61 participants (59.8%) stated an age range of 16–18 years to be appropriate, in line with legal age of consent “*I would say when you reach an age where you can make your own medical decisions whilst understanding the consequences, 16–18.”* However, there was a wide range of suggested ages from 7 to 21 years.

### Sample donation and data handling

**Fig. 1:**
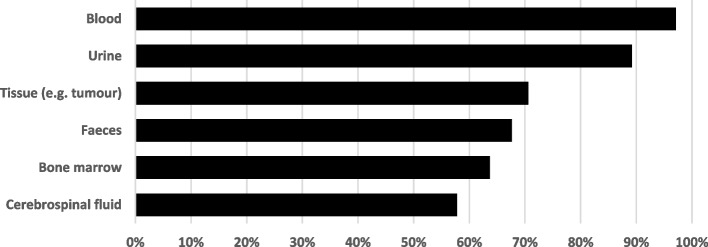
Acceptability (%) for sample donation by sample type

Almost all participants were comfortable donating blood (97.1%, *N* = 99) and urine (89.2%, *N* = 91) (Fig. 1). Participants felt the main reasons CYP might hesitate to donate samples were privacy (55.6%, *N* = 40/72) “*People might worry about their personal data being lost or stolen.*”, personal views (37.5%, *N* = 27/72) e.g. *“[I] don’t like the idea of my tissue being kept and used for long period of time and not knowing what it is used for”* and embarrassment “*Donating faeces can make many people feel uncomfortable”* and ethical views (23.6%, *N* = 17/72): “*I would only donate samples if I knew exactly how they would be used, in line with my own personal views.”*

If CYP donate biospecimens to a biobank, the majority of participants agree that medical details could be stored alongside the sample (82.2%, *N* = 83). They acknowledge that their donated samples were more valuable when linked to additional clinical data, “*The information will help make the sample more useful so I would be happy providing extra details”.*

The minority who had reservations about additional information being stored, would be satisfied if data is stored without personal identifiers. “*There should be absolutely no identifying information, or any information that is not strictly related to you [sic] biological medical record”* and “*as long as the government doesn't make blood-tracing nanobots that could find me, then I'm fine.”*

One-fifth of participants are not interested in following their samples *“[I] don’t really mind what the clever clogs do with it”.* The majority of participants (81.2%, *N* = 81) would like to know more about how their samples are used but are pragmatic in their requests, *“It would be interesting to know but if it is more work/expenses for hospitals *etc. *I don’t mind not knowing*” suggesting the “*Ability to opt in to receiving updates on research”.*

## Discussion

This study provides insights from UK CYP on consent procedures and ethical issues surrounding participation in research involving biobanks. It adds valuable data in a field where there is little literature on CYP perspectives.

In our surveyed population we demonstrate CYP have positive attitudes towards participation in research involving biobanks, and acceptability of both prospective and deferred consent is high. Altruism, sample frugality and bodily integrity were important themes for CYP, concurrent with views of adults [23, 24], and CYP participating in critical care trials [21]. We acknowledge that there is hesitancy in a minority of CYP to donate, due to needle phobia, pain, or aversion towards the bodily fluid to be donated. While we expected needle phobia and pain to be main barriers to donation, we were surprised by the young people’s hesitancy to donate stool samples, due to perceived embarrassment.

In line with previous studies involving the views of practitioners [15] and parents [14], CYP were receptive to deferred consent, provided it was obtained appropriately [15]. This is a marked change, compared to a similar small unpublished survey conducted in our centre previously, in which CYP from the same region were less receptive to deferred consent. Post-hoc, we identified two potential factors may have influenced this, although these were not specifically mentioned by the participants.

First, the coronavirus disease 2019 (COVID-19) pandemic. The pandemic introduced viral testing on a large scale to the general population, including CYP. During this time, media outlets frequently reported on the scientific progress made into this novel disease, utilizing data generated from COVID-19 swabs taken from the general public. This introduced CYP to a surrogate process of donating biospecimens to biobanks for research and what impact this contribution might have. Having experienced donating biospecimens, attitudes to aforementioned factors might have changed.

Second, in 2020, the new organ donation law in England came into effect, changing the opt-in to an opt-out system [27]. The opt-out system can effectively be seen as a proxy deferred consent, as it assumes every adult is happy to be an organ donor, unless they actively record a decision to be excluded from the donor programme. We postulated that this may have raised awareness of different forms of consent practices nationally. Deferred consent for biobanking assumes consent to take biospecimens with a retrospective option to decline for the sample to be used. The reasoning behind this option is to reduce the number of sample attempts (i.e. blood draws) to minimise pain and distress in children and young people.

The right to assent is protected by the United Nations Convention on the Rights of the Child, article 12, which states: “*States Parties shall assure to the child who is capable of forming his or her own views the right to express those views freely in all matters affecting the child, the views of the child being given due weight in accordance with the age and maturity of the child”* [28]. Our results clearly show that CYP should be actively involved in the consent procedure, by giving assent in conjunction with their parents, or by being approached for reconsent once they are old enough. Some participants provided accounts of negative personal experiences in hospital and not being involved or listened to, in order to emphasize the importance of CYP participation in their own healthcare and maintaining autonomy. The benefit and need for CYP with capacity to assent has become increasingly clear [29–33]. This is consistent with the attitude that children have grown more independent within the home, school, and medical setting [34], and increasingly seen as autonomous individuals [35]. There are concerns that CYP might lack capacity to give meaningful consent particularly around the complex concept of biobanking [21, 36]. However, there is evidence that even adults lack a deeper understanding of biobanking [23]. McGregor and Ott [37] demonstrated the capacity of adolescents/young adults (aged 12–24 years) to consent is similar to that of adults. Therefore, aiding CYP to provide meaningful informed consent is a pivotal task of the consenting researcher, and should be achievable in most circumstances.

CYP are largely supportive of having medical data stored alongside their biobanked sample and recognized the value of data and risks of data sharing. Due to widespread social media use, CYP are used to providing consent and understanding their risks and benefits of sharing their personal data. CYP with long-term conditions using health technology to manage their illness [38] have highlighted such concerns. Concerns about governance and entrusting their sample to a biobank are legitimate in light of the Alder Hey organ scandal, involving the removal of human tissue and organs of hundreds of deceased children without the knowledge and consent of their parents between 1988 and 1996. Following public inquiry [39], this led to the Human Tissue Act 2004 [40] and new recommendations for consent approaches, to prevent this from happening again [41].

Research requires trust from those willing to participate. It is vital CYP are provided with sufficient, comprehensive information tailored to their age and expected level of understanding. It is important that clear and transparent information on privacy, data and sample handling is available and clearly communicated.

### Limitations

Although this study adds to the paucity of community CYP perspectives on consent procedures and ethical issues surrounding biobanking, it has limitations. Most respondents were from the North East of England, and their views might not represent those in the UK as a whole. This study was conducted in a high-income country, and there is currently no evidence to support whether our results might be applicable to low- or middle-income country settings or, other cultural settings. Specific ethical challenges have been reported surrounding the ethical adequacy for large-scale biobanking and sample use during disease outbreaks, for example during the 2014 Ebola outbreak in Africa [42], that are rarely seen in the setting of high-income countries.

Additionally, we acknowledge that most of our participants are older and female CYP, and the perspectives could change if we had a larger proportion of younger CYP. The current number of younger participants was too low to conduct a subset analysis.

The survey allowed for opinions and perspectives to be shared by the participants, but our data lacks the in-depth detail you could get from an interview-based approach.

## Conclusions

For CYP in the UK prospective and deferred consent are highly acceptable in the context of biobanking.

CYP should be included in consent procedures for biobank participation, when they reach capacity.

Important themes surrounding consent are altruism, frugality, body integrity and ownership.

Clear communication and justification of the need for biobanking is paramount to ensure willingness to participate in research involving biobanking.

This study highlights the lack of clear guidelines and information around consent procedures for biobanking of samples from children and young persons. We would suggest that national bodies need to develop these in conjunction with active engagement and input from CYP themselves.

## Supplementary Information

**Additional file 1.**

## Data Availability

The datasets generated and analysed for this study are available from the corresponding author upon reasonable request.

## Abbreviations

CYP: Children and young people
DIAMONDS: Diagnosis and Management of Febrile Illness using RNA Personalised Molecular Signature Diagnosis
UK: United Kingdom
YPAGne: Young Person’s Advisory Group North England
