# Supplementary material for: Biobanking and consenting to research: a qualitative thematic analysis of young people’s perspectives in the North East of England
Source: BMC Med Ethics. 2023 Jul 5;24:47. doi: 10.1186/s12910-023-00925-w (PMC10324191; doi:10.1186/s12910-023-00925-w)
Supplement: Supplementary file 1 — Additional file 1. [file 12910_2023_925_MOESM1_ESM.docx]

**Additional File 1: Questionnaire**

**Biobank Questionnaire**

Thank you for helping us. We believe it is important to find out what people think about medical issues.

We would like to ask you opinion about storing samples in a biobank.

Biobanking is when we store samples of blood or tissue, and data for use later, either for medical or scientific research.

You do not need to take part in this survey, only if you choose to (and thanks again!).

Any information you give to us will be stored anonymously (without your name so it cannot be trace to you).

*Required

**Section 1 – Participant information**

1. How old are you?* (choose one)
   - 12-15 years
   - 16-18 years
   - Over 18
   - Other: _________________________
2. What gender do you identify as?* (choose one)
   - Male
   - Female
   - Prefer not to say
   - Other: _________________________
3. What is the first part of you postcode? (e.g. NE2, NE35)*

Participant experience with biobanking

We would like to know how much you know about biobanking beforehand

1. Have you heard of biobanking before?*
   - Yes
   - No
2. Have you ever had any previous experience of biobanking, personally or with someone close to you? If yes, would you like to tell us about your experience
3. Have you ever had an overnight stay in hospital?*
   - I have never had to stay overnight in hospital
   - I have had overnight stays for short minor illnesses
   - I have had stays to help manage a chronic illness(es)
   - Other: _______________________

**Section 2**

We are going to give you a few cases where biobanking might be used. We want to know your opinion. There are no right or wrong answers, please tell us as much as you want.

Case 1 – You require a routine medical or surgical procedure (like giving blood or having a small operation such as having a skin lesion cut off). How would you feel if beforehand you were asked to give an extra sample of blood or tissue for a biobank? *

I would strongly disagree to this O O O O I would strongly agree to this

Please explain you answer/leave any comments about case 1*

Case 2 - You are rushed into hospital in an emergency. A sample of blood is taken for a diagnostic test. When they take your blood there may be some left over. How would you feel if the extra blood was taken for a biobank, and you were asked afterwards if this was okay? This is called deferred consent, and if you said no the sample would be destroyed. *

I would strongly disagree to this O O O O I would strongly agree to this

Please explain you answer/leave any comments about case 2*

Case 3 - your parent or guardian tells you that when you were a baby, they consented to a sample of your blood being stored in a biobank for research. Do you think it is important for you to be asked at a later age whether you give your own permission for your samples to be used in the future? *

- Yes
- No

Case 3 continued - If yes, at what age should this be? Please tell us your opinions and thoughts

Do you think children and young people should also be involved in giving consent to biobanking samples where possible (i.e. not just their parents)?*

- Yes
- No
- Maybe

Please tell us your thoughts and opinions on children and young people being involved in giving consent to biobanking samples*

What kind of samples would you be happy to donate to a biobank if they were being collected anyway for a necessary medical or surgical procedure? (Please select all that apply)*

- Blood
- Urine
- Faeces
- Cerebrospinal fluid – the fluid that coats your brain and spinal cord, taken using a needle in your back
- Tissue e.g. sample of a tumour, sample of muscle, sample of bowel wall

If there are any samples you would not be happy to donate, please explain your thoughts and opinions*

If you were happy for a sample to be stored and used in a biobank, what extra information would you be happy to be stored about you as the donor? This information helps researchers and makes your sample more useful but shouldn't be enough to identify you. (Please select all that apply)*

- No further information
- Your age
- Your postcode
- Your medical details
- Other: ___________________________

Please tell us more about your thoughts and opinions on the information you would be happy to be stored about you with your sample

Are there any reasons you might hesitate to donate your samples? (Please select all that apply)

- Personal views
- Ethical views
- Religion
- Privacy
- Other: __________________________

If your sample was used for research, would you like to be informed?

- Yes
- No
- Maybe

Please tell us your thoughts and opinions about how you might like to find out about your sample being used and what information you would want to know.
